# Stability of SARS-CoV-2 Spike G614 Variant Surpasses That of the D614 Variant after Cold Storage

**DOI:** 10.1128/mSphere.00104-21

**Published:** 2021-03-31

**Authors:** Sheng-Yu Huang, Yu-An Kung, Peng-Nien Huang, Sheng-Yun Chang, Yu-Nong Gong, Yi-Ju Han, Huan-Jung Chiang, Kuan-Ting Liu, Kuo-Ming Lee, Chia-Yu Chang, Chia-Ching Chang, Chung-Guei Huang, Shin-Ru Shih

**Affiliations:** a Research Center for Emerging Viral Infections, College of Medicine, Chang Gung University, Taoyuan, Taiwan; b Department of Medical Biotechnology and Laboratory Science, College of Medicine, Chang Gung University, Taoyuan, Taiwan; c Division of Infectious Diseases, Department of Pediatrics, Linkou Chung Chang Gung Memorial Hospital, Taoyuan, Taiwan; d Bachelor Program in Artificial Intelligence, College of Engineering, Chang Gung University, Taoyuan, Taiwan; e Department of Laboratory Medicine, Linkou Chang Gung Memorial Hospital, Taoyuan, Taiwan; f Graduate Institute of Biomedical Science, Division of Biotechnology, College of Medicine, Chang Gung University, Taoyuan, Taiwan; g Department of Biological Science and Technology, College of Biological Science and Technology, National Yang Ming Chiao Tung University, Hsinchu, Taiwan; h Center for Intelligent Drug Systems and Smart Bio-devices (IDS2B), National Yang Ming Chiao Tung University, Hsinchu, Taiwan; i Department of Electrophysics, National Yang Ming Chiao Tung University, Hsinchu, Taiwan; j Institute of Physics, Academia Sinica, Nankang, Taipei, Taiwan; k Research Center for Chinese Herbal Medicine, Chang Gung University of Science and Technology, Taoyuan, Taiwan; l Research Center for Food and Cosmetic Safety, Chang Gung University of Science and Technology, Taoyuan, Taiwan; m Graduate Institute of Health Industry Technology, College of Human Ecology, Chang Gung University of Science and Technology, Taoyuan, Taiwan; National Institute of Allergy and Infectious Diseases

**Keywords:** SARS-CoV-2, D614G mutation, spike protein, RNA integrity, temperature, ACE2, D614 variant, cold storage, stability

## Abstract

Severe acute respiratory syndrome coronavirus 2 (SARS-CoV-2) carrying the D614G mutation on the spike protein is the predominant circulating variant and is associated with enhanced infectivity. However, whether this dominant variant can potentially spread through the cold chain and whether the spike protein affects virus stability after cold storage remain unclear. To compare the infectivity of two SARS-CoV-2 variants, namely, SARS-CoV-2 variants with spike protein with the D614 mutation (S-D614) and G614 mutation (S-G614), after different periods of refrigeration (4°C) and freezing (−20°C). We also determined the integrity of the viral RNA and the ability of the spike protein to bind angiotensin-converting enzyme 2 (ACE2) after storage at these conditions. The results showed that SARS-CoV-2 was more stable and infectious after storage at −20°C than at 4°C. Particularly, the S-G614 variant was found to be more stable than the S-D614 variant. The spike protein of the S-G614 variant had better binding ability with the ACE2 receptor than that of the S-D614 variant after storage at −20°C for up to 30 days. Our findings revealed that SARS-CoV-2 remains stable and infectious after refrigeration or freezing, and their stability and infectivity up to 30 days depends on the spike variant. Stability and infectivity are related to each other, and the higher stability of S-G614 compared to that of S-D614 may contribute to rapid viral spread of the S-G614 variant.

**IMPORTANCE** It has been observed that variants of severe acute respiratory syndrome coronavirus 2 (SARS-CoV-2) are more stable and infectious after storage at −20°C than at 4°C. A SARS-CoV-2 S-D614G variant is currently the most dominant variant in circulation and is associated with enhanced infectivity. We compared the stability of two SARS-CoV-2 variants: the early S-D614 variant carrying the D614 spike protein and the new S-G614 variant carrying the G614 spike protein, stored at both 4°C and −20°C for different periods. We observed that SARS-CoV-2 remains stable and infectious after refrigeration or freezing, which further depends on the spike variant, that is, the ability of the spike protein to bind with the ACE2 receptor with higher efficiency. The high stability of the S-G614 variant also explains its rapid spread and infectivity. Therefore, precautions should be taken during and after handling food preserved under cold conditions.

## INTRODUCTION

In 2019, a new human coronavirus, severe acute respiratory syndrome coronavirus 2 (SARS-CoV-2), emerged in Wuhan, China (1), and the disease it causes has been named coronavirus disease 2019 (COVID-19) by the World Health Organization. As of 9 March 2021, over 117 million COVID-19 cases have been recorded worldwide, causing more than 2.6 million deaths with a fatality rate of 0.8% to 14.5% (2). The estimated basic reproductive number for SARS-CoV-2 is approximately 2 to 6, whereas that for SARS-CoV is only 0.19 to 1.08 (3), which explains the rapid global spread of SARS-CoV-2 (4–7). Because COVID-19 cases are continuously increasing, the control of SARS-CoV-2 spread is of utmost importance.

Since June 2020, news reports from several countries have speculated that seafood products that are transported to the market through a cold chain may have caused the wide spread of the virus (8). Additionally, several studies have indicated that SARS-CoV-2 cannot remain viable at high temperatures (10–12), suggesting that SARS-CoV-2 could be transmitted through the cold chain. However, these studies examined only the changes in the infectious SARS-CoV-2 titer (50% tissue culture infective dose [TCID_50_]) of the early variant carrying spike protein with the D614 mutation (S-D614).

The SARS-CoV-2 spike protein is a receptor binding protein that is critical in its infectivity. A SARS-CoV-2 S-D614G variant emerged in February 2020 in Europe and is currently the most dominant variant in circulation (13). The reproductive number of the SARS-CoV-2 S-D614G variant increased by 31% compared with that of the wild-type SARS-CoV-2 S-D614 (14). The rapid spread of the S-D614G variant quickly garnered attention and necessitated investigations to understand its effect on SARS-CoV-2 infectivity. The S-D614G variant showed no significant association with disease severity and no change in sensitivity to neutralizing antibodies compared with the wild type (13–15). However, the titers, fitness, and transmission of S-G614-carrying SARS-CoV-2 are higher than those of the S-D614-carrying SARS-CoV-2 in hamster and human upper respiratory cells (15, 16). Although there is a clear difference in titers, the mechanism underlying these differences between the mutants remains unclear. Particularly, how mutations affect the binding ability of the virus to angiotensin-converting enzyme 2 (ACE2), an entry receptor for SARS-CoV-2, is unclear.

To compare the stability of early SARS-CoV-2 variants carrying the D614 spike protein (S-D614 variant) and new SARS-CoV-2 variant carrying the G614 spike protein (S-G614 variant), we stored both variants at 4°C and −20°C for different periods (up to 30 days). We then analyzed the effects of these temperatures on infectious viral titers, viral RNA integrity, and the ability of the virus to bind to ACE2. Our study provides insights into the differences in viral infectivity after exposure to different temperatures.

## RESULTS

### Higher infectivity of the SARS-CoV-2 S-G614 variant than that of S-D614 after cold storage.

**FIG 1:**
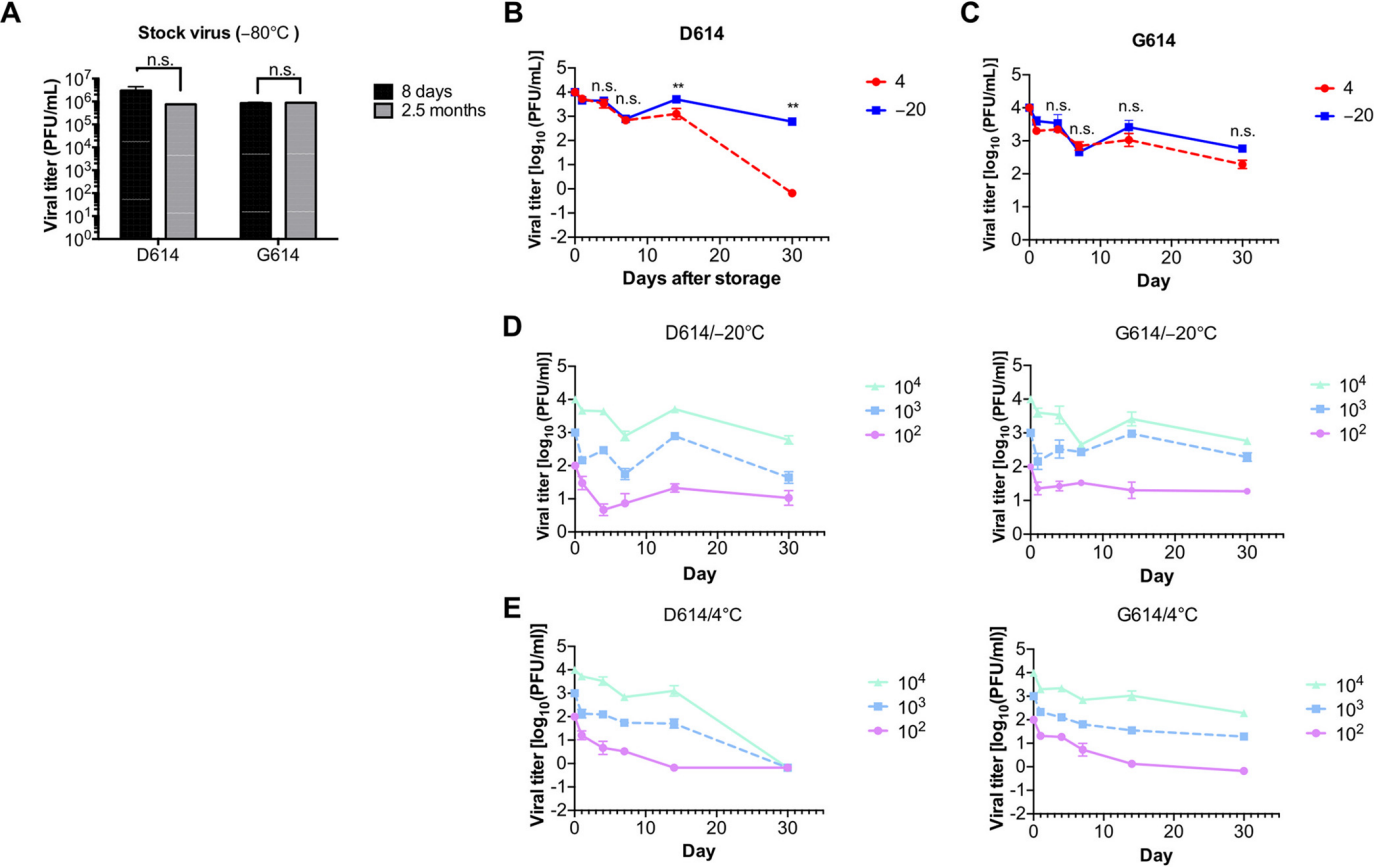
Titers of severe acute respiratory syndrome coronavirus 2 (SARS-CoV-2) S-D614 and S-G614 variants after storage at 4°C, −20°C, and −80°C for different durations. (A) Titers of virus stocks stored at −80°C for 8 days and 2.5 months. (B and C) Comparison of viral titers of the S-D614 (D614) (B) and S-G614 (G614) (C) variants. The stocks of SARS-CoV-2 variants were diluted to 10^4^ PFU/ml and stored at 4°C and −20°C for different durations. (D) Comparison of viral titers of the S-D614 and S-G614 variants stored at 4°C and −20°C for 30 days, with initial viral concentrations of 10^4^ PFU/ml. (E and F) Titers of the S-D614 and S-G614 variants of SARS-CoV-2 with different initial viral concentrations stored at 4°C (E) or −20°C (F) for different durations. Error bars represent the standard deviations (SD) of three independent experiments. Statistical significance was determined by conducting an unpaired *t* test (*n *= 3; *, *P* < 0.05; **, *P* < 0.01; ns, not significant).

To determine the effect of temperature on the S-D614 and S-G614 variants of SARS-CoV-2, we evaluated the titers of the two variants after cold storage. The titers of the SARS-CoV-2 S-D614 and S-G614 variants remained stable after 2.5 months of storage at −80°C compared with the baseline values (8 days of storage) (Fig. 1A). However, 14 days of storage at 4°C significantly reduced the titer of the S-D614 variant compared with storage at −20°C (Fig. 1B). The titers of the S-D614 variant were almost undetectable after 30 days of storage at 4°C but decreased by only 1 log unit relative to the baseline level (0 days of storage) when stored at −20°C. These findings indicate that the S-D614 variant is more stable at −20°C than at 4°C.

Similarly, the titers of the S-G614 variant decreased approximately 1 log unit compared with the initial titer after storage at −20°C for 30 days (Fig. 1C). Interestingly, unlike the S-D614 variant, the S-G614 variant retained a considerable degree of infectivity when stored at 4°C for 30 days (Fig. 1C), suggesting that the stability of the S-G614 variant after 30-day storage at 4°C and −20°C is similar.

Next, we determined the stability of the variants at low initial viral titers, because the viral concentrations in the environment and on surfaces are typically not as high as that in laboratory cultures. For this purpose, we compared higher initial titers (10^4^) in Fig. 1B and C with lower initial titers (10^2^ and 10^3^) in Fig. 1D and E. S-D614 and S-G614 with both high and low initial titers remained infectious even after 30 days of storage at −20°C (Fig. 1D). However, the S-D614 variant exhibited higher infectivity for a longer duration at high initial viral titers (10^3^ and 10^4^ PFU/ml) than low initial titers (10^2^ PFU/ml) at 4°C (Fig. 1E). Notably, the relatively stable S-G614 variant with high initial titers remained infectious when stored at 4°C for 30 days (Fig. 1E). These findings indicate that SARS-CoV-2 with low viral titers is less stable at 4°C.

### Higher RNA integrity of the SARS-CoV-2 S-G614 variant than that of S-D614 after cold storage.

**FIG 2:**
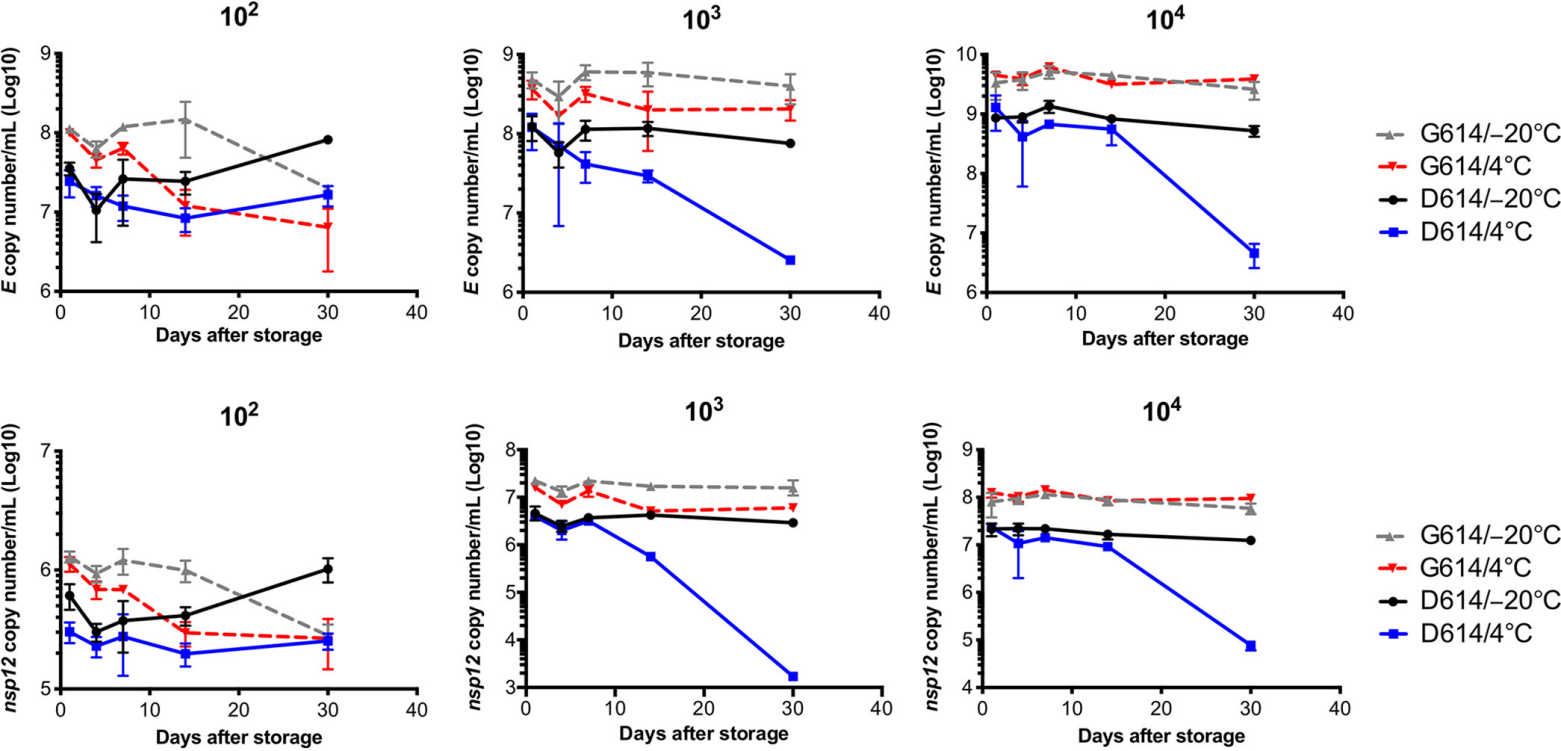
Severe acute respiratory syndrome coronavirus 2 RNA copy numbers of the S-D614 and S-G614 variants stored at 4°C, −20°C, and −80°C. Virus stocks were diluted to 10^2^, 10^3^, and 10^4^ PFU/ml and stored at 4°C and −20°C for different durations. Viral RNA was extracted, and copy numbers were determined by targeting *E* (top) and *nsp12* (bottom) genes. Error bars represent the standard deviations (SD) derived from three independent experiments.

We explored the reasons for the decrease in viral titers after storage. First, we determined whether SARS-CoV-2 RNA is degraded upon storage. The TaqMan probe system was used to quantify the expression of structural *E* and nonstructural *nsp12* genes because degraded viral RNA cannot be amplified and detected (17). In both *E* and *nsp12* genes (Fig. 2), the trends in the copy numbers of viral RNA from the S-D614 and S-G614 variants were consistent with those of the infectious viral titers. Therefore, we speculated that the decrease in viral titer correlates with viral RNA degradation.

**FIG 3:**
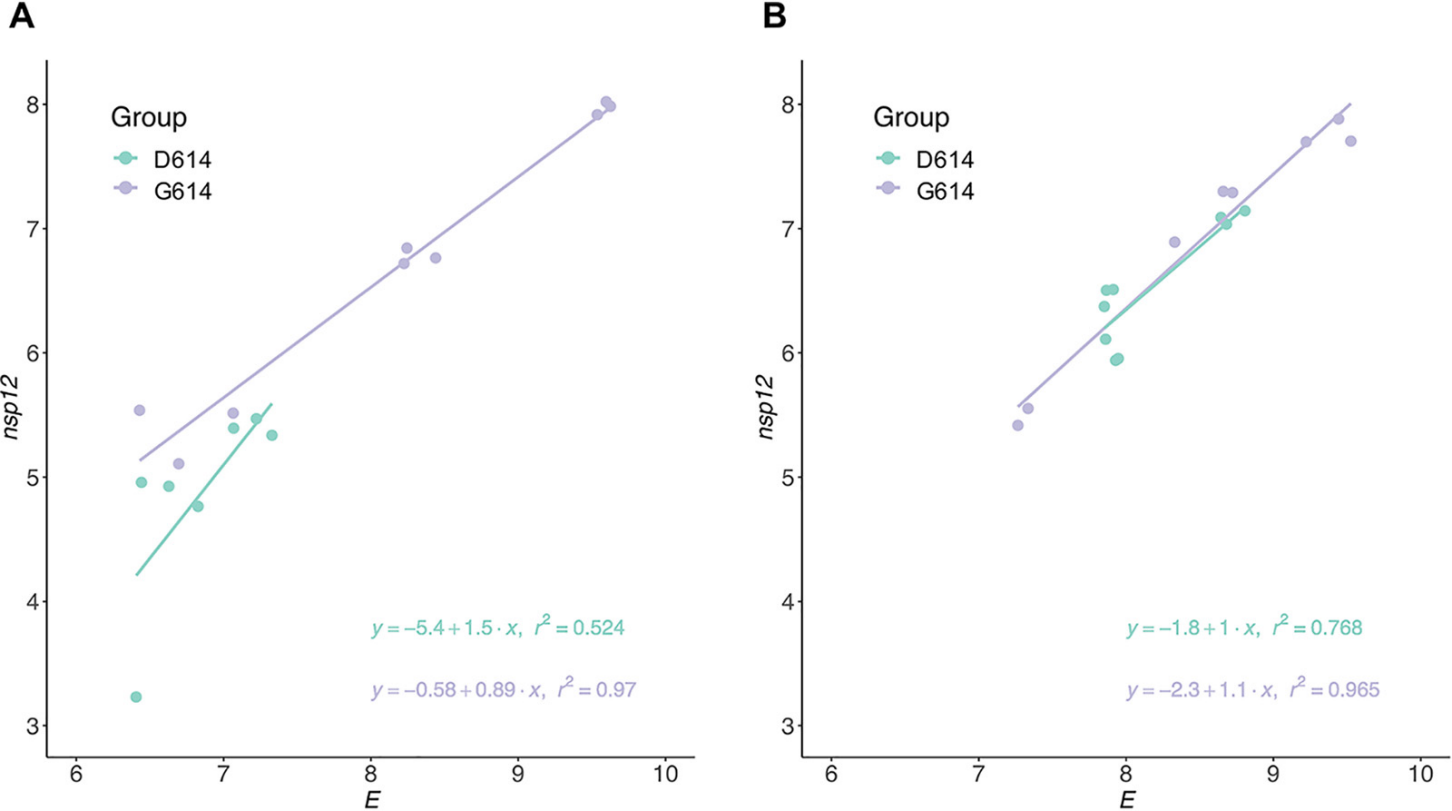
Integrity of SARS-CoV-2 genomic RNA. (A and B) Correlation between the copy number of *nsp12* and *E* genes in the S-D614 and S-G614 variants of SARS-CoV-2 stored at 4°C (A) or −20°C (B) for 30 days. To increase the sample size to enable better comparison, we pooled samples of the same variant that were stored at the same temperature. The respective regression equations and *r*^2^ values are shown.

To confirm this hypothesis, we analyzed the integrity of SARS-CoV-2 RNA by determining the linear relationship between *nsp12* and *E* gene copies as previously described (17). The S-G614 variant showed a higher correlation between the *E* and *nsp12* genes than the S-D614 variant after storage at 4°C and −20°C (Fig. 3), indicating that the genome of the S-G614 variant is more stable. Moreover, the correlation between *E* and *nsp12* genes of the S-D614 variant was higher at −20°C than at 4°C, whereas that in the S-G614 variant was similar at both temperatures. These results are consistent with those observed for viral titers (Fig. 1B to E).

### Greater binding of the SARS-CoV-2 S-G614 variant to recombinant human ACE2 than that of S-D614 after cold storage.

**FIG 4:**
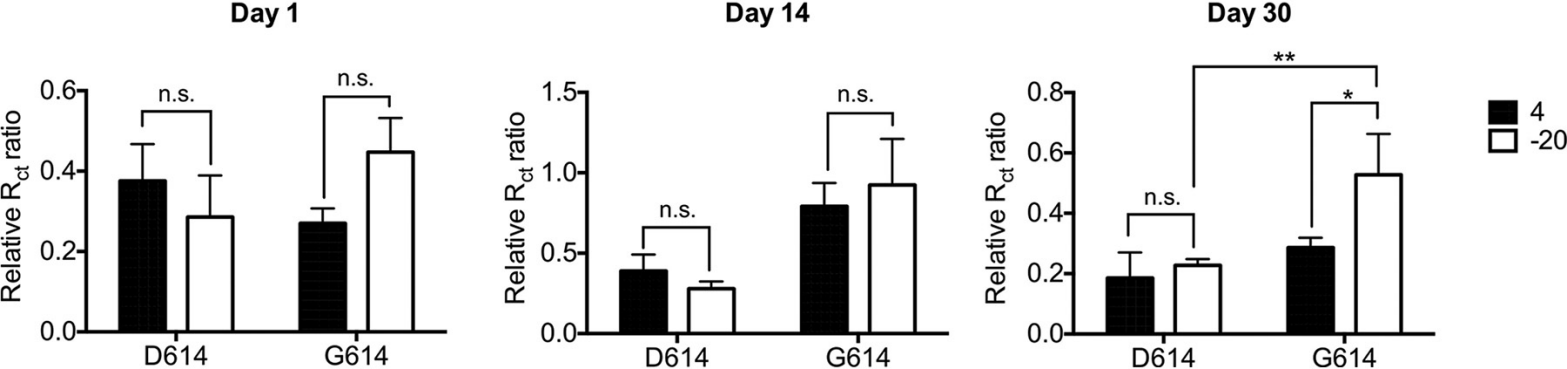
Binding of the SARS-CoV-2 spike protein with recombinant human angiotensin-converting enzyme 2 (rhACE2). Pseudoviruses carrying SARS-CoV-2 S-D614 and S-G614 were stored at −20°C or 4°C for different durations, and then added to rhACE2-coated chips. The relative *R*_Ct_ ratio was considered proportional to the amount of spike protein bound to ACE2. Error bars represent standard deviations derived from three independent experiments. Significant differences were analyzed using the two-way ANOVA with Newman-Keuls multiple-comparison tests of data. (*n *= 3; *, *P* < 0.05; **, *P* < 0.01; ns, not significant).

Next, we explored the difference in the recombinant human ACE2 (rhACE2)-binding ability of the S-D614 and S-G614 variants after storage at different temperatures. We coated Pd nano-thin film polyethylene terephthalate electrodes with rhACE2 and then added either S-D614 or S-G614 pseudovirus at equal transduction unit (transduction unit [TU]/μl) to the chip and observed the changes in resistance. We found no significant difference in the binding ability of the S-D614 and S-G614 pseudoviruses to rhACE2 at different temperatures on the first day (Fig. 4). Notably, the binding ability of the S-D614 pseudovirus at different temperatures did not significantly change after 30 days of storage. However, after 14 and 30 days, the binding ability of the S-G614 pseudovirus at −20°C was significantly higher than that at 4°C. Thus, the rhACE2-binding ability of the S-G614 variant on day 30 of storage at −20°C was significantly higher than that of the S-D614 variant. Moreover, storage at 4°C was not conducive for the maintenance of the rhACE2-binding ability of both variants, whereas storage at −20°C better maintained the binding ability of the S-G614 variant compared with that of the S-D614 variant.

## DISCUSSION

Several studies have discussed the stability of SARS-CoV-2 at different temperatures or environmental conditions. To our knowledge, this is the first study to report the differences in viral infectivity after exposure to different temperatures by studying viral RNA integrity and binding ability of the spike protein to rhACE2, along with viral titer. Our findings also provided a possible explanation for the rapid spread of the S-G614 variant; it may be attributed to its higher stability.

The stability of viruses is affected by the concentration of the viral stock, and the viral titer present in the inoculum affects viral infectivity. The effects of temperature on SARS-CoV-2 have been previously investigated (12, 18, 19). However, the initial viral titer used was higher than 10^6^ half-maximal tissue culture infectious dose/ml (equivalent to ∼10^7^ PFU/ml). They found that the viral titer was quite stable, in contrast to our finding. We believe that the difference in the results is mainly because the initial virus concentration used is different from that in our study (10^2^ to 10^4^ PFU/ml), 3 × 10^5^ to 3 × 10^3^ times higher. Our results in Fig. 1D and E show that the higher the virus concentration, the higher the stability of the virus, and the reduction in viral titer at 4°C is more significant than that at −20°C and −80°C. Our data, based on relatively low virus concentrations that are close to the actual concentration of viral particles in the environment, showed that the initial viral titer also affects its stability. La Scola et al. (20) reported that no virus culture was obtained from samples with cycle threshold (*C_T_*) values of ≥34 after the amplification of *E* gene, suggesting that patients with *C_T_* values of ≥34 are no longer infectious and can be discharged. A *C_T_* value of 34 for the *E* gene is equivalent to 6.16 log_10_ copies/ml of SARS-CoV-2. Although all viral nucleic acids are not infectious viral particles, the expression of the viral RNA copies or the *C_T_* values can provide an indication as to whether SARS-CoV-2 variants remain infectious after 1 month of storage at 4°C or −20°C. Our results indicated that SARS-CoV-2 in refrigerated or frozen foods exhibit considerable stability and infectivity even after 30 days. Notably, the aforementioned studies used the early SARS-CoV-2 S-D614 variant; in contrast, we studied both early and new variants and found that the new SARS-CoV-2 S-G614 variant is more stable than S-D614. Interestingly, the S-G614 variant we used had three other nonsynonymous mutations in the coding region and one mutation in its untranslated region. Whether these mutations contribute to the enhanced integrity of the SARS-CoV-2 G614 variant genome is unexplored. In addition, we analyzed the binding ability of the spike protein and rhACE2 through Pd nano-thin film polyethylene terephthalate electrodes. It has been shown that ACE2 undergoes glycation modification leading to integration into the plasma membrane (21). However, we conducted protein-protein interaction *in vitro*, and therefore, we believe that the expression of ACE2 without glycosylated modification through Escherichia coli will not affect the performance of binding ability with spike protein.

In conclusion, we found that the S-D614 variant does not remain infectious after storage at 4°C for 30 days but remains infectious after 30 days of storage at −20°C. Notably, the currently prevalent G614 mutation-harboring variant remains infectious regardless of the storage temperature for 30 days. Thus, SARS-CoV-2 may transmit through foods preserved under cold conditions. Therefore, precautions should be taken while eating and handling foods preserved under cold conditions. Moreover, although the susceptibility to serum/antibody neutralization of the S-G614 variant remains unchanged, its high stability still plays an important role in its ability to spread.

## MATERIALS AND METHODS

### Cell line.

VeroE6 (American Type Culture Collection, Manassas, VA, USA) cells were grown in Dulbecco’s modified Eagle’s medium (DMEM) containing 10% fetal bovine serum (FBS), 1% antibiotic/antimycotic solution, and 1% l-glutamine (Gibco, Grand Island, NY, USA). The cells were cultured at 37°C with 5% CO_2_.

### Viruses.

The S-D614 SARS-CoV-2/human/TWN/CGMH-CGU-01/2020 (GISAID [https://www.gisaid.org] accession number EPI_ISL_411915; NCBI accession number MT192759.1) and S-G614 SARS-CoV-2/human/TWN/CGMH-CGU-25/2020 (GISAID accession number EPI_ISL_444278; NCBI accession number MT479227.1) variants were originally isolated from a patient with COVID-19 from Chang Gung Memorial Hospital. Viral amplification and manipulation were performed in an accredited biosafety level 3 laboratory at the Chang Gung Memorial Hospital. The S-G614 variant has four synonymous and four nonsynonymous mutations in the coding region. The nonsynonymous mutations include ORF1ab-C794T(T265I), ORF1ab-C14144T(P4715L), S-A1841G(D614G), and ORF3a-G171C(Q57H). There is also a mutation in the 5′ untranslated region—C241T. The SARS-CoV-2 stocks of the S-D614 and S-G614 variants were diluted in DMEM with 2% FBS at 10^2^ to 10^4^ PFU/ml and stored at 4°C and −20°C for different durations (1, 4, 7, 21, and 30 days). Thereafter, they were transferred to −80°C until further analyses such as titration, RNA quantification, and RNA integrity analysis. Viruses were propagated in Vero E6 cells, maintained in DMEM with 2% FBS and stored at −80°C until further study.

### Plaque assay.

VeroE6 cells were seeded into a six-well cell culture plate and grown for 18 to 24 h at 37°C. At 90% confluence, the medium was removed, and 10-fold serial viral dilutions (from 10^0^ to 10^−4^) were allowed to adsorb onto the cells for 60 min. The cells were washed with phosphate-buffered saline and grown in DMEM containing 2% FBS with 0.4% of agarose for 3 days. To visualize the plaques, the cells were inactivated with 10% formalin for at least 1 h and were then stained with 0.5% crystal violet.

### Viral RNA extraction and real-time PCR (RT-PCR).

Viral RNA was extracted using the LabTurbo Viral DNA/RNAMini kit according to the manufacturer’s instructions and using the LabTurbo 48 Compact System (Taigen Bioscience, Taipei, Taiwan). The *E* and *nsp12* genes were quantified as described previously (17).

### SARS-CoV-2 pseudovirus.

The SARS-CoV-2-S Luc pseudovirus (provided by the National RNAi Core Facility, Academia Sinica, Taiwan) uses pCMVdeltaR8.91 and pcDNA3.1 to express the spike protein on the viral surface. The transfer vector pLAS2w.FLuc.Ppuro carried by the virus expresses firefly luciferase.

### Recombinant expression and characterization of the human ACE2 protein.

rhACE2 was expressed in the Escherichia coli system as previously described (22–24). rhACE2 refolding was confirmed by Western blotting, and its interaction with the SARS-CoV-2 spike protein receptor-binding domain was confirmed by electrochemical impedance spectroscopy measurements as described below.

### Electrochemical impedance spectroscopy measurements.

The electrochemical properties of the ACE2-functionalized Pd nano-thin film polyethylene terephthalate electrodes were determined based on our previous study protocol (22, 25).

### Statistical analysis.

We used Student’s *t* test to compare the results of the viral titer changes between 4°C and −20°C on the same day, presented in Fig. 1B and C. The two-way analysis of variance (ANOVA) with Newman-Keuls multiple-comparison test was used to analyze the ACE2-binding ability of the same virus variant at different temperatures and that of different variant viruses at the same temperature. Statistical analysis was performed using GraphPad Prism v9.3.3 (GraphPad Software, Inc., CA, USA). Data are presented as means ± standard errors of means. Results with a *P* value of <0.05 were considered statistically significant. Linear regression models were used to determine the correlation between genome copies of structural (*E*) and nonstructural genes (*nsp12*) with C*_T_* values obtained from RT-PCR, and the *R^2^* value was used to assess model fitness. Statistical analysis was conducted using the R software v3.6.1 (26), and the distribution of genome copies and their correlations were visualized using the R package ggplot2 as previously described (17, 27).

## References

[1] Huang C, Wang Y, Li X, Ren L, Zhao J, Hu Y, Zhang L, Fan G, Xu J, Gu X, Cheng Z, Yu T, Xia J, Wei Y, Wu W, Xie X, Yin W, Li H, Liu M, Xiao Y, Gao H, Guo L, Xie J, Wang G, Jiang R, Gao Z, Jin Q, Wang J, Cao B. 2020. Clinical features of patients infected with 2019 novel coronavirus in Wuhan, China. Lancet 395:497–506. doi:10.1016/S0140-6736(20)30183-5. 31986264 PMC7159299

[2] Johns Hopkins University of Medicine. 2020. Mortality analyses. Coronavirus Resource Center, Johns Hopkins University of Medicine, Baltimore, MD. https://coronavirus.jhu.edu/data/mortality. Accessed 29 December 2020.

[3] Chowell G, Castillo-Chavez C, Fenimore PW, Kribs-Zaleta CM, Arriola L, Hyman JM. 2004. Model parameters and outbreak control for SARS. Emerg Infect Dis 10:1258–1263. doi:10.3201/eid1007.030647. 15324546 PMC3323341

[4] Rahman B, Sadraddin E, Porreca A. 2020. The basic reproduction number of SARS-CoV-2 in Wuhan is about to die out, how about the rest of the world? Rev Med Virol 30:e2111. doi:10.1002/rmv.2111. 32431085 PMC7267092

[5] Sanche S, Lin YT, Xu C, Romero-Severson E, Hengartner N, Ke R. 2020. High contagiousness and rapid spread of severe acute respiratory syndrome coronavirus 2. Emerg Infect Dis 26:1470–1477. doi:10.3201/eid2607.200282. 32255761 PMC7323562

[6] Wang L, Wang Y, Ye D, Liu Q. 2020. Review of the 2019 novel coronavirus (SARS-CoV-2) based on current evidence. Int J Antimicrob Agents 55:105948. doi:10.1016/j.ijantimicag.2020.105948. 32201353 PMC7156162

[7] Lai CC, Shih TP, Ko WC, Tang HJ, Hsueh PR. 2020. Severe acute respiratory syndrome coronavirus 2 (SARS-CoV-2) and coronavirus disease-2019 (COVID-19): the epidemic and the challenges. Int J Antimicrob Agents 55:105924. doi:10.1016/j.ijantimicag.2020.105924. 32081636 PMC7127800

[8] Zhao Y. 19 June 2020. China’s CDC experts investigate Xinfadi market three times, announce groundbreaking virus tracing discovery. Global Times, Beijing, People’s Republic of China. https://www.globaltimes.cn/content/1192146.shtml.

[9] Reference deleted

[10] Riddell S, Goldie S, Hill A, Eagles D, Drew TW. 2020. The effect of temperature on persistence of SARS-CoV-2 on common surfaces. Virol J 17:145. doi:10.1186/s12985-020-01418-7. 33028356 PMC7538848

[11] Kratzel A, Steiner S, Todt D, V'kovski P, Brueggemann Y, Steinmann J, Steinmann E, Thiel V, Pfaender S. 2020. Temperature-dependent surface stability of SARS-CoV-2. J Infect 81:452–482. doi:10.1016/j.jinf.2020.05.074. PMC7832219 32504748

[12] Biryukov J, Boydston JA, Dunning RA, Yeager JJ, Wood S, Reese AL, Ferris A, Miller D, Weaver W, Zeitouni NE, Phillips A, Freeburger D, Hooper I, Ratnesar-Shumate S, Yolitz J, Krause M, Williams G, Dawson DG, Herzog A, Dabisch P, Wahl V, Hevey MC, Altamura LA. 2020. Increasing temperature and relative humidity accelerates inactivation of SARS-CoV-2 on surfaces. mSphere 5:e00441-20. doi:10.1128/mSphere.00441-20. 32611701 PMC7333574

[13] Korber B, Fischer WM, Gnanakaran S, Yoon H, Theiler J, Abfalterer W, Hengartner N, Giorgi EE, Bhattacharya T, Foley B, Hastie KM, Parker MD, Partridge DG, Evans CM, Freeman TM, de Silva TI, Sheffield COVID-19 Genomics Group, McDanal C, Perez LG, Tang H, Moon-Walker A, Whelan SP, LaBranche CC, Saphire EO, Montefiori DC. 2020. Tracking changes in SARS-CoV-2 spike: evidence that D614G increases infectivity of the COVID-19 virus. Cell 182:812–827.e19. doi:10.1016/j.cell.2020.06.043. 32697968 PMC7332439

[14] Leung K, Pei Y, Leung GM, Lam TT, Wu JT. 2020. Empirical transmission advantage of the D614G mutant strain of SARS-CoV-2. medRxiv. doi:10.1101/2020.09.22.20199810. PMC8662801 34886945

[15] Plante JA, Liu Y, Liu J, Xia H, Johnson BA, Lokugamage KG, Zhang X, Muruato AE, Zou J, Fontes-Garfias CR, Mirchandani D, Scharton D, Bilello JP, Ku Z, An Z, Kalveram B, Freiberg AN, Menachery VD, Xie X, Plante KS, Weaver SC, Shi PY. 2020. Spike mutation D614G alters SARS-CoV-2 fitness. Nature doi:10.1038/s41586-020-2895-3. PMC8158177 33106671

[16] Hou YJ, Chiba S, Halfmann P, Ehre C, Kuroda M, Dinnon KH, Leist SR, Schäfer A, Nakajima N, Takahashi K, Lee RE, Mascenik TM, Graham R, Edwards CE, Tse LV, Okuda K, Markmann AJ, Bartelt L, de Silva A, Margolis DM, Boucher RC, Randell SH, Suzuki T, Gralinski LE, Kawaoka Y, Baric RS. 2020. SARS-CoV-2 D614G variant exhibits efficient replication ex vivo and transmission in vivo. Science 370:1464–1468. doi:10.1126/science.abe8499. 33184236 PMC7775736

[17] Huang CG, Lee KM, Hsiao MJ, Yang SL, Huang PN, Gong YN, Hsieh TH, Huang PW, Lin YJ, Liu YC, Tsao KC, Shih SR. 2020. Culture-based virus isolation to evaluate potential infectivity of clinical specimens tested for COVID-19. J Clin Microbiol 58:e01068-20. doi:10.1128/JCM.01068-20. 32518072 PMC7383522

[18] Chan KH, Sridhar S, Zhang RR, Chu H, Fung AY-F, Chan G, Chan JF-W, To KK-W, Hung IF-N, Cheng VC-C, Yuen K-Y. 2020. Factors affecting stability and infectivity of SARS-CoV-2. J Hosp Infect 106:226–231. doi:10.1016/j.jhin.2020.07.009. 32652214 PMC7343644

[19] Chin AWH, Chu JTS, Perera MRA, Hui KPY, Yen HL, Chan MCW, Peiris M, Poon LLM. 2020. Stability of SARS-CoV-2 in different environmental conditions. Lancet Microbe 1:e10. doi:10.1016/S2666-5247(20)30003-3. 32835322 PMC7214863

[20] La Scola B, Le Bideau M, Andreani J, Hoang VT, Grimaldier C, Colson P, Gautret P, Raoult D. 2020. Viral RNA load as determined by cell culture as a management tool for discharge of SARS-CoV-2 patients from infectious disease wards. Eur J Clin Microbiol Infect Dis 39:1059–1061. doi:10.1007/s10096-020-03913-9. 32342252 PMC7185831

[21] Schrom E, Huber M, Aneja M, Dohmen C, Emrich D, Geiger J, Hasenpusch G, Herrmann-Janson A, Kretzschmann V, Mykhailyk O, Pasewald T, Oak P, Hilgendorff A, Wohlleber D, Hoymann HG, Schaudien D, Plank C, Rudolph C, Kubisch-Dohmen R. 2017. Translation of angiotensin-converting enzyme 2 upon liver- and lung-targeted delivery of optimized chemically modified mRNA. Mol Ther Nucleic Acids 7:350–365. doi:10.1016/j.omtn.2017.04.006. 28624211 PMC5423349

[22] Chang CY, Huang YT, Chang PC, Su CH, Hsu KC, Li X, Wu CH, Chang CC. 2019. Surface active flexible palladium nano-thin-film electrode development for biosensing. Inorg Chem Commun (Camb) 107:107461. doi:10.1016/j.inoche.2019.107461.

[23] Chang CC, Su YC, Cheng MS, Kan LS. 2002. Protein folding by a quasi-static-like process: a first-order state transition. Phys Rev E Stat Nonlin Soft Matter Phys 66:021903. doi:10.1103/PhysRevE.66.021903. 12241210

[24] Chang CC, Cheng MS, Su YC, Kan LS. 2003. A first-order-like state transition for recombinant protein folding. J Biomol Struct Dyn 21:247–256. doi:10.1080/07391102.2003.10506920. 12956608

[25] Chang CY, Chen W, Su CH, Chang PC, Huang YT, Hsu KC, Yuan CJ, Chang CC. 2019. Enhanced bioconjugation on sputtered palladium nano-thin-film electrode. Appl Phys Lett 114:93702. doi:10.1063/1.5087030.

[26] R Development Core Team. 2019. R: a language and environment for statistical computing. R Foundation for Statistical Computing, Vienna, Austria.

[27] Wickham H. 2016. ggplot2: elegant graphics for data analysis. Springer-Verlag, New York, NY.

